# Genetic and genomic analysis of the seed-filling process in maize based on a logistic model

**DOI:** 10.1038/s41437-019-0251-x

**Published:** 2019-07-29

**Authors:** Shuangyi Yin, Pengcheng Li, Yang Xu, Jun Liu, Tiantian Yang, Jie Wei, Shuhui Xu, Junjie Yu, Huimin Fang, Lin Xue, Derong Hao, Zefeng Yang, Chenwu Xu

**Affiliations:** 1 Jiangsu Provincial Key Laboratory of Crop Genetics and Physiology, Co-Innovation Center for Modern Production Technology of Grain Crops, Key Laboratory of Plant Functional Genomics of Ministry of Education, Yangzhou University, 225009 Yangzhou, China; 2 Jiangsu Yanjiang Institute of Agricultural Sciences, 226541 Nantong, China

**Keywords:** Agricultural genetics, Quantitative trait

## Abstract

Seed filling is a dynamic process that determines seed size and nutritional quality. This time-dependent trait follows a logistic (S-shaped) growth curve that can be described by a logistic function, with parameters of biological relevance. When compared between genotypes, the filling dynamics variations are explained by the differences of parameter values; as such, the parameter estimates can be considered as “traits” for genetic analysis to identify loci that are associated with the seed-filling process. We carried out genetic and genomic analysis of the seed-filling process in maize, using a recombinant inbred line (RIL) population derived from the two inbred lines with contrasting seed-filling dynamics. We recorded seed dry weight at 14 time points after pollination, spanning the early filling phases to the late maturation stages. Fitting these data to a logistic model allowed for estimating 12 characteristic parameters that can be used to meaningfully describe the seed-filling process. Quantitative trait locus (QTL) mapping of these parameters identified a total of 90 nonredundant loci. Using bulked segregant RNA-sequencing (BSR-seq) analysis, we identified eight genes that showed differential gene expression patterns at multiple time points between the extreme pools, and these genes co-localize with the mapped QTL regions. Two of the eight genes, *GRMZM2G391936* and *GRMZM2G008263*, are implicated in starch and sucrose metabolism, and biosynthesis of secondary metabolites that are well known for playing a vital role in seed filling. This study suggests that the logistic model-based approach can efficiently identify genetic loci that regulate dynamic developing traits.

## Introduction

Maize is a widely cultivated crop in the world, providing an important source for food, feed, and biofuels. Increasing and stabilizing maize productivity has been a primary goal of breeders. Among many agronomic traits that are associated with yield potential, the seed-filling process is an important factor that affects seed size and the final yield (Takai et al. 2005; Sadras and Egli 2008; Borrás et al. 2009; Eichenberger et al. 2015). Seed filling is a complex trait involving cell expansion and accumulation of proteins, oils, and carbohydrates. Despite being under genetic control, this developmental process is also highly sensitive to environmental fluctuations, such as heat and drought stresses. Optimizing the rate and duration of seed filling can maximize partitioning of photosynthates and assimilates to the seed, reduce the negative effects of potential adverse environments in the late growing season, and consequently ensure a high and stable yield. Therefore, a thorough understanding of the genetic and environmental factors regulating seed filling will assist breeding efforts to develop high-yielding varieties with improved resilience capacity to abiotic stresses.

Seed filling in maize can be divided into multiple successive and interrelated developmental stages, beginning with successful pollination and initiation of seed development and ending when the seeds are physiologically mature. The dynamic feature of the trait poses a significant challenge for quantitative genetic analysis, because at individual developmental stages or time points, different sets of genes are involved. Most of the current studies focused only on the linear growth phase by measuring the dry seed weight over an isometric time period to estimate the filling rate (Maydup et al. 2010; Liu et al. 2011; Zhang et al. 2016; Carmo-Silva et al. 2017) Although this approach allowed for rough estimation of the average filling rate over a specific period of the filling duration, it failed to capture the dynamic developing features of the entire filling process, such as the maximum filling rate and the time to reach the maximum filling rate.

A better strategy for studying such a time-dependent trait is to fit a growth curve to the phenotypic values across the entire developmental process and analyze the fitted parameters of the growth trajectory using quantitative genetic approaches. The effectiveness of this strategy has been proven by combining genetic analysis and an ecophysiological model to reveal the response of maize leaf growth to temperature and water deficit (Reymond et al. 2003). For the physiological process of seed filling, the more common approach is to separate the entire process into the segmented model, according to different phases of seed development (Gambín et al. 2008; Alvarez Prado et al. 2013, 2014). This segmented model usually indicates physiological maturity based on the intersection of the second and third phases (Alvarez Prado et al. 2013, 2014). However, seed maturity is programmed and not abruptly reached. Therefore, this approach based on the segmented model lost dynamic information about the continuous seed-filling process (Vega and Sadras 2003; Borrás et al. 2009). Considering the above case, it is a need to use a nonlinear function to model the dynamic process. Logistic models have been widely used to analyze complex dynamic developing traits, such as human population growth (Ershkov 2011; Peckham et al. 2018), plant height (Sun and Frelich 2011), and the leaf biomass in forest stands (Ogawa 2012). Meanwhile, seed development follows a typical sigmoid growth curve that can be described by a logistic function with a few fitted parameters. Instead of analyzing many “traits” derived from phenotypic measurements at multiple time points, genetic analysis can be limited to a few parameters with biological relevance (Thornley et al. 2007; Yin et al. 2018).

In this study, we performed genetic and genomic analysis of the seed-filling process in maize, using a recombinant inbred line (RIL) population derived from the two inbred lines (DH1M and T877) that differed significantly in their seed-filling dynamics. For phenotyping, we measured seed dry weight at multiple time points after pollination, from the early filling phases to the late maturation stages. By fitting these data to a logistic model, we estimated 12 characteristic parameters that can be used to explain the seed-filling dynamics. Quantitative trait locus (QTL) mapping of these parameters identified abundant nonredundant QTL loci, revealing a complex nature of the seed-filling process. Using bulked segregant RNA-sequencing (BSR-seq) analysis, we further identified eight genes that showed differential gene expression patterns at multiple time points between the extreme pools, and these genes co-localize with the mapped QTL regions. Two of the eight genes, *GRMZM2G391936* and *GRMZM2G008263*, are implicated in starch and sucrose metabolism, and biosynthesis of secondary metabolites that are well known for playing a vital role in seed filling. This study suggests that the logistic model-based approach can efficiently identify genetic loci that regulate dynamic developing traits.

## Materials and methods

### Plant materials, field experiments, and phenotyping

The RIL population consisting of 208 lines was derived from the cross of the parental genotypes DH1M and T877 that differed significantly in the rate and duration of grain filling. DH1M had a shorter grain-filling duration and produced smaller grains, while T877 had a much longer grain-filling period and produced much larger grains. The experiments were conducted at three geographically different locations in China: Nantong (N31°55′, E121°37′) in 2015, Yangzhou (N32°22′, E119°16′) in 2016, and Sanya (N18°23′, E109°44′) in 2017. For each RIL, a total of 78 plants were grown in a plot consisting of six rows, with a row length of 3.0 m, a distance between rows of 0.5 m, and 13 plants per row. The pollination dates were recorded for individual RIL lines to determine the sampling time. Seeds were sampled at 10, 15, 20, 25, 30, 35, 40, 43, 46, 49, 52, 55, 58, and 61 days after pollination (DAP). At each time point, two ears with the synchronous developmental process were selected. Fifty seeds in the middle of each ear were removed and dried to a constant weight at 70–80 °C after fixing at 105 °C for 1 h. The dry weight of 50 seeds was measured and recorded.

### Growth curve fitting and parameter estimation

The relationship between the seed dry weight (*w*) and the number of days after pollination (*t*) was described by a logistic function in the following form (West et al. 2001):

1 $$w = \frac{k}{{1 + ae^{ - bt}}}$$

where *k*, *a*, and *b* are fitted parameters. *k* estimates the final or upper limit of seed dry weight; *b* is related to the filling rate; and *a* is associated with both the rate and duration of seed filling.

The instantaneous rate of seed filling (*v*) at the moment of *t* was obtained by taking the first derivative of formula (1):

2 $$v = \frac{{dw}}{{dt}} = \frac{{kabe^{ - bt}}}{{(1 + ae^{ - bt})^2}}$$

Based on this equation, the average filling rate ($\bar v$) and the active filling duration (*T*) were also estimated; $\bar v$ was calculated from the integral of the formula (2) and *T* was obtained by *k*/$\bar v$.

**Fig. 1:**
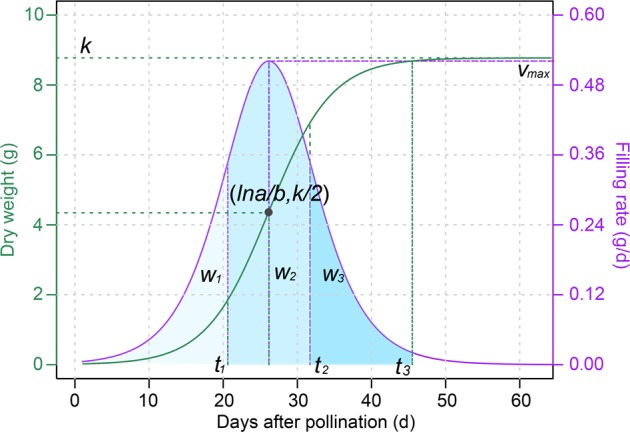
A diagrammatic representation of the seed-filling curve (green) and the filling rate curve (purple). Several seed-filling characteristic parameters are indicated. *k* is the final seed dry weight; *v*_max_ is the maximum filling rate; *lna/b* is time for reaching the median final dry weight; and *w*_1_, *w*_2_, and *w*_3_ are accumulated dry weight during the gradual growth period (*t*_1_), fast growth period (*t*_2_–*t*_1_), and slow growth period (*t*_3_–*t*_2_), respectively. *t*_1_ and *t*_2_ are DAPs (days after pollination) reaching the first and second inflexion of the filling curve, respectively; and *t*_3_ is the total filling duration

According to formulas (1) and (2), the distributions of seed dry weight (*w*, green color) and filling rate (*v*, purple color) over time *t* are presented in Fig. 1. By taking the second derivative of formula (1) and setting it equal to zero, an inflexion point (*lna/b*, *k/2*) on the seed-filling curve was defined; *lna/b* is the time for reaching the median final dry weight (*k*/2) and at this time, the maximum instantaneous filling rate (*v*_max_) is reached.

By taking the second derivative of formula (2) and setting it equal to zero, two inflexion points were defined at the time points *t*_1_ and *t*_2_. The stage from fertilization to *t*_1_ is defined as the lag or gradual growth period and that between *t*_1_ and *t*_2_ is defined as the fast growth period. Theoretically, seed dry weight will never be able to reach the asymptotic maximum *k*. In this study, seed filling was considered to be complete when *w* = 0.99*k*. Based on this assumption, an estimate of the length of the seed-filling duration (*t*_3_) was calculated by substituting 0.99*k* for *w* in formula (1). The stage from *t*_2_ to *t*_3_ is defined as the slow growth period or the maturation phase. As shown under the filling rate curve, the accumulated dry weight during each of the three filling periods, denoted as *w*_1_, *w*_2_, and *w*_3_, respectively, can also be calculated.

In summary, a total of 12 characteristic parameters related to the dynamic seed-filling process were estimated. The symbols of these parameters together with their calculation formulas and biological definitions are listed in Table 1. Parameters *k*, *a*, and *b* were estimated using a nonlinear least-squares approach implemented in R (Bates and Watts 1988).

**Table 1:** Characteristic parameters related to seed filling in maize

Parameter symbol	Biological significance	Formula
*k*	Final seed dry weight (g)	
*b*	Relative filling rate	
*lna/b*	Time for median final dry weight (DAP)	
*t*_1_	Time for first inflexion of filling rate (DAP)	$t_{\it{1}} = - \frac{{{\it{ln}}\left( {\frac{{2 + \sqrt 3 }}{a}} \right)}}{b}$
*t*_2_	Time for second inflexion of filling rate (DAP)	$t_{\it{2}} = - \frac{{{\it{ln}}\left( {\frac{{2 - \sqrt 3 }}{a}} \right)}}{b}$
*t*_3_	Filling duration (DAP)	$t_{\it{3}} = \frac{{{\it{ln}}(99a)}}{b}$
*w*_1_	Accumulation during gradual growth period (g)	$w_{\it{1}} = \frac{k}{{3 + \sqrt 3 }}$
*w*_2_	Accumulation during fast growth period (g)	$w_{\it{2}} = \frac{{\sqrt 3 }}{3}k$
*w*_3_	Accumulation during slow growth period (g)	$w_{\it{3}} = \left( {\frac{{49}}{{100}} - \frac{{\sqrt 3 }}{6}} \right)k$
$\bar v$	Average filling rate (g/DAP)	$\bar v = \frac{{kb}}{6}$
*T*	Active filling period (DAP)	$T = \frac{6}{b}$
*v*_max_	Maximum filling rate (g/DAP)	$v_{max} = \frac{{kb}}{4}$

### Statistical analysis of the parameters

In this study, we fitted one filling curve for each inbred line in an environment and estimated a list of characteristic parameters for each inbred line. Calculating the descriptive statistics of the parameters was performed in R. Broad-sense heritability (*H*^2^) across multiple environments was estimated as $H^{\mathrm{2}} = \delta _g^2/(\delta _g^2 + \delta ^2/e)$ (Knapp et al. 1985), where $\delta _g^2$ is the genetic variance, *δ*^2^ is the error variance, and *e* is the number of environments. Pearson correlation coefficients between characteristic parameters were calculated using the *agricolae* software package (De Mendiburu 2014). The best linear unbiased prediction values of *v*_max_ were calculated based on the combined data from three environments using *lmer* function in the *lme4* package (Bates et al. 2015) in R. In this model, genotypes and environments were considered as random factors.

### Genotyping and bin-map construction

DNA was extracted from the young healthy leaves of 208 RILs and two parents. Genotyping was performed with the Illumina MaizeSNP50 BeadChip, which contained over 56,110 evenly distributed single-nucleotide polymorphisms (SNPs) covering the entire maize genome. Chi-square tests were conducted for all SNPs to detect segregation distortion. SNPs with a segregation distortion test *P* < 0.001 or containing abnormal bases were filtered out. To eliminate redundant markers, we used a sliding-window approach (Huang et al. 2009) to construct a bin map. The order of bin markers were then checked using the *ripple* function in the *qtl* package (Broman et al. 2003). Genetic distances between bin markers were calculated using the “Kosambi” function (Kosambi 1944).

### QTL analysis

QTLs associated with the 12 characteristic parameters were mapped using composite interval mapping (CIM) with the *lmem.qtler* package in R (Gutierrez et al. 2016). For CIM, we used a window size of 10 cM, a step size of 1 cM, and a threshold that was estimated through a Bonferroni correction based on the effective number of markers (*M*_eff_) (Li and Ji 2005). In this study, the *M*_eff_ was 35 and the threshold was 2.8.

### Differential gene expression and enrichment analysis

Based on the *v*_max_ values estimated from the combined data obtained from 2015 to 2017, we selected two pools of inbred lines (13 lines per pool) that displayed contrasting seed-filling rates but reached *v*_max_ at a similar time, ~30 days after pollination. Moreover, the difference significance of characteristic parameters between two pools was detected using the *t.test* function in R. These lines along with the two parents were grown at Yangzhou, China in the summer of 2017, using the same experimental design as described above. At 30 days post pollination, seeds were collected and mRNAs were then isolated from individual plants. Two bulked mRNA samples were then created by pooling the mRNAs of all individuals in each pool. The pooled mRNA samples were subjected to RNA-seq analysis to identify genes that were differentially expressed between the two pools. In the RNA-seq analysis process, we first standardized the read counts using TMM algorithm (Robinson and Oshlack 2010). Under the biological coefficient of variation was equal to 0.2, we performed the genewise exact test for differences between two pools of negative binomial counts using the *exactTest* function in the *edgeR* software package (Robinson et al. 2009). After Bonferroni correction, the genes with adjusted *P* values less than 0.05 and an absolute value of logarithm fold change greater than two were identified as differentially expressed genes (DEGs). To determine the putative biological functions of DEGs, Gene Ontology (GO), and Kyoto Encyclopedia of Genes and Genomes (KEGG) enrichment analyses were performed using the *clusterProfiler* package (Yu et al. 2012). The GO terms and KEGG pathways with adjusted *P* values < 0.05 through false discovery rate (FDR) (Hochberg 1995) correction were identified as significant.

### Detection of the associated SNPs

To identify SNPs that could distinguish between the two bulks, a series of tests were performed in R. Fisher’s exact tests were first performed for each SNP followed by calculation of the SNP index of each bulk and *G* value of each SNP using the *QTLseqr* package (Mansfeld and Grumet 2018). Through these procedures, the ΔSNP index per site was then acquired. Using 0.05 as a threshold after FDR correction, the significantly associated SNPs were identified. DEGs with significantly associated SNPs and enriched terms or pathways were considered as associated genes.

### Expression analysis of the associated genes

To further characterize the expression of the associated genes in the filling process, seeds of parents and two extreme inbred lines were sampled at 10, 20, 30, 40, and 50 DAP. At each time point, seeds in the middle of two similar ears were sampled as two biological repeats, followed by RNA isolation and RNA-seq analysis. The reads per kilobase of exon per million mapped reads (FPKM) of individual genes at different time points were calculated based on the length of the genes and read counts. In addition, the dry weights of 50 seeds for each inbred line at different time points were measured to fit the seed growth curves.

## Results and discussion

### Fitting the seed-filling curves using a logistic function and estimating the parameters

**Fig. 2:**
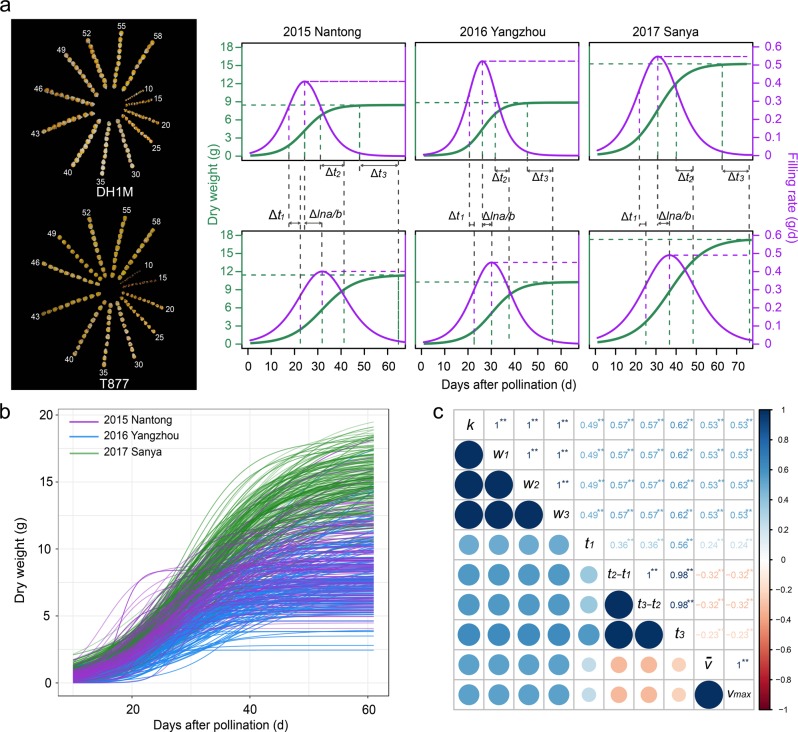
Seed-filling curves of the two parents and RILs, and correlations among the parameter estimates of seed dry weight, filling duration, and filling rate. **a** On the left are seed changes of DH1M and T877 during filling process. Seed-filling curves (green) and filling rate curves (purple) of DH1M and T877 in three different environments are on the right. **b** Seed-filling curves of RILs across three different environments. Purple, blue, and green curves are filling curves of RILs in Nantong (2015), Yangzhou (2016), and Sanya (2017), respectively. **c** Correlations among parameter estimates of seed dry weight, filling duration, and filling rate. Blue color represents a positive correlation and red color represents a negative correlation; color depth indicates the level of correlation. * and ** represent significant correlation at *P* = 0.05 and 0.01 levels, respectively

The RIL population consisting of 208 lines was derived from the parental genotypes DH1M and T877 that differed in the final seed dry weight and rate, and duration of seed filling. In particular, DH1M produced smaller seeds and had a shorter filling duration and a higher filling rate, while T877 produced larger seeds and had a longer filling period and a lower rate of filling (Fig. 2a). These plants were grown in three different environments and seed dry weight was measured at various time points from pollination to maturity. A logistic function was used to characterize the seed-filling process of both parents and RILs (Fig. 2b). The average determination coefficients (*R*^*2*^) of the fitting curves in three environments were 0.95, 0.95, and 0.97, respectively, indicating a good fit of the logistic curve to the seed-filling data. We estimated a total of 12 parameters that are associated with seed dry weight accumulation, seed-filling rate, and filling duration (Table S1). There were significant variations between genotypes, environments, and strong genotype-by-environment interactions for the observed data and all the parameter estimates. The variation coefficients of the parameters ranged from 9.53 to 45.11% among the RIL lines, and the estimated *H*^2^ of the parameters ranged from 69.26 to 79.33%. These data suggested the presence of significant genetic variation for seed-filling dynamics in the segregating population. Compared with results based on the segmented model (Alvarez Prado et al. 2013, 2014), heritabilities related to seed weight were similar. However, heritabilities related to filling duration were evidently larger in this study.

Pearson’s correlation analysis revealed a positive relationship between the total filling duration (*t*_3_) and the final seed dry weight (*k*) and between the duration (*t*_1_, *t*_2_–*t*_1_, and *t*_3_–*t*_2_) and the accumulated dry weight in each of the three filling phases (*w*_1_, *w*_2_, and *w*_3_), respectively (Fig. 2c). On average, the three phases contributed ~21%, 58%, and 20% to the final dry weight, respectively. The final seed dry weight was also positively correlated with the parameters associated with filling rate ($\bar v$, and *v*_max_). In general, the filling duration estimates were negatively associated with those of filling rate; interestingly however, *t*_1_, the duration of the early or lag phase of seed development was positively associated with the filling rate parameters. Several other studies also suggested the importance of this phase in regulating the seed growth rate in the following seed-filling phase in soybeans and maize (Egli and Wardlaw 1980; Egli et al. 1989; Jones et al. 1984). The lag phase is dominated by cell division and cell number determination; thus, this phase likely mediates final seed size by regulating the endosperm sink capacity and this capacity in turn governs the dry matter deposition rate in endosperm cells during the subsequent seed-filling stage.

### Genetic map construction and QTL mapping

For genetic mapping of QTLs associated with the parameters in the RIL population, we constructed a high-density linkage map consisting of 3227 bin markers (Fig. S1). Summary statistics of the map are presented in Table S2. The number of bin markers per chromosome varied from 111 to 503. The total length of the linkage map was 2450 cM, with chromosome 1 being the longest and chromosome 2 the shortest. The average genetic distance between two adjacent markers was 0.76 cM; chromosome 5 had the highest marker density and chromosome 2 had the lowest marker density. A heatmap showing the linkage and recombination relationship between bin markers is illustrated in Fig. S2a, and a grid for the haplotypes of the RIL population is shown in Fig. S2b.

**Fig. 3:**
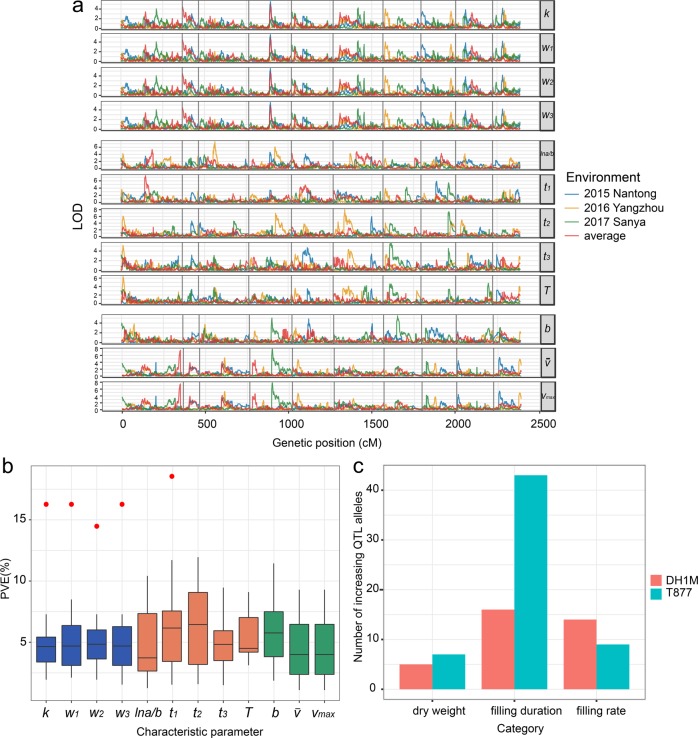
QTL mapping of 12 characteristic parameters. **a** LOD curves of 12 characteristic parameters in different environmental conditions. Some peaks of the same characteristic parameter in one environment do not appear in another environment. **b** Phenotypic contribution rates of QTLs associated with the 12 characteristic parameters. Blue, orange, and green colors represent PVE (phenotypic variability explained) related to dry weight, filling duration, and filling rate, respectively. Red points indicate the higher PVEs. **c** Number of increasing QTL alleles in different categories contributed by each parent

QTL analysis of the estimated parameters was performed, based on the data obtained from individual environments, as well as combined data from all three environments. For analysis of combined data, the average values of the parameters were calculated. These analyses identified a total of 90 nonredundant QTLs associated with 12 parameters (Tables S3 and S4). In particular, 12 QTLs were associated with accumulation of seed dry weight (*k*, *w*_1_, *w*_2_, and *w*_3_); 23 QTLs were related to filling rate (*b*, $\bar v$, and *v*_max_); and 60 QTLs were linked to seed-filling duration (*T*, *t*_1_, *t*_2_, *t*_3_, and *lna/b*). Because of significant genotype-by-environment interactions, certain QTLs identified in one environment were not detected in another environment (Fig. 3a). Across three environments, the mean percentage of phenotypic variation explained by individual QTLs ranged from 4.59 to 6.29% (Fig. 3b and Table S3). Both parents contributed favorable alleles at various loci; however, T877 provided more increasing QTL alleles for seed dry weight and filling duration, while DH1M conferred more increasing QTL alleles associated with filling rate (Fig. 3c). This latter observation is consistent with the phenotypic differences observed between the two parents. In addition, we identified some pleiotropic QTL (Table S4), such as QTL located at 14.1 cM chromosome 7 controlled both seed dry weight and filling duration. This case was also found by other studies based on independent time points (Zhang et al. 2016) or the segmented model (Alvarez Prado et al. 2014). This similar result indicated that the correlated traits were likely to be controlled by common genetic mechanism, which helped explaining the phenotypic correlation. Furthermore, the locus located at 56.3 cM on chromosome 2 was associated with seed dry weight, which was consistent with other studies (Austin and Lee 1998; Alvarez Prado et al. 2013). The loci mapped at 95.4−104.1 cM on chromosome 5 co-localized with QTL related to seed weight in other studies (Guo et al. 2011; Alvarez Prado et al. 2013). The locus detected at 43 cM on chromosome 7 was located in the same region as other reports related to seed weight and filling rate (Li et al. 2012).

### Bulked segregant RNA-seq analysis of the RIL population

**Fig. 4:**
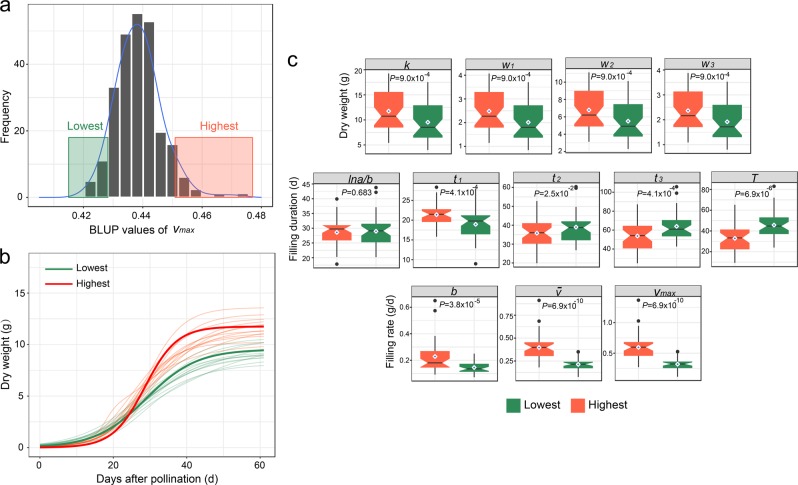
Construction of two extreme pools based on *v*_max_. **a** The distribution of *v*_max_ and selection intervals of two extreme pools were also indicated. **b** The filling curves of RILs in two extreme pools (transparent lines) and the average filling curves of lowest and highest pools (solid lines). **c** Comparisons of estimates of 12 characteristic parameters between the two extreme pools. Red color represents the highest pool and green color represents the lowest pool

To complement QTL mapping, we carried out a genomic analysis to identify genes that regulate the seed-filling dynamics using a BSR-seq approach. For this purpose, we selected two pools of RILs that showed extreme phenotypes of *v*_max_, the maximum rate of seed filling (Fig. 4a). The rationale of this strategy was based on the observation that *v*_max_ was positively correlated to the final seed weight. One of the pools consisted of 13 RILs with high-*v*_max_ values of 0.451–0.472, while another pool contained 13 RILs with low-*v*_max_ values of 0.421–0.428. The selected lines were planted and evaluated at Yangzhou in 2017. The fitting curves derived from individual RILs and combined analysis of individual pools are shown in Fig. 4b. It is apparent that the two pools differed in their seed-filling dynamics; accordingly, the parameter estimates are statistically different except for the parameter *lna/b*, the time when *v*_max_ is reached (Fig. 4c). Such analysis further validated the previous conclusions that the lag phase duration *t*_1_ was positively correlated with the filling rate and that the filling rate was positively associated with seed dry weight accumulation.

**Fig. 5:**
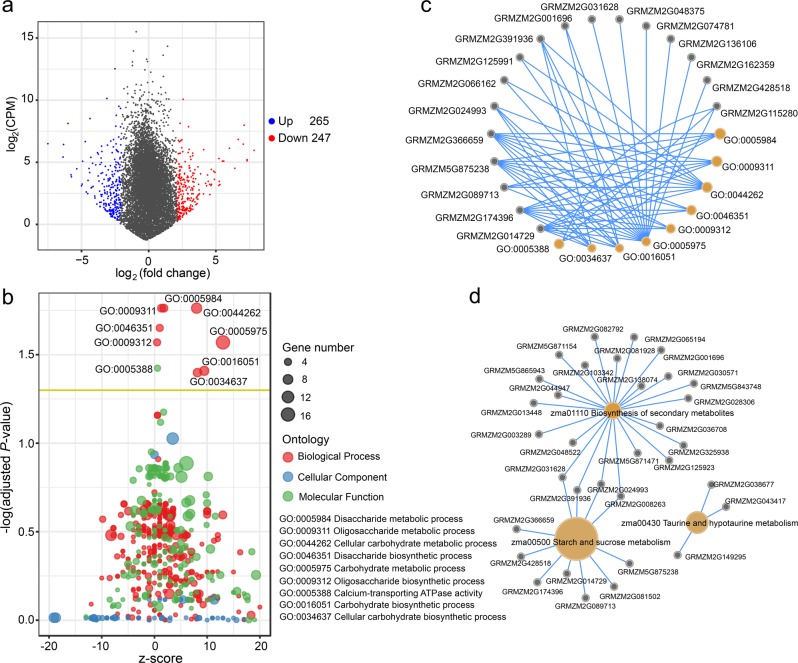
Enrichment analysis of differentially expressed genes between the two extreme pools. **a** A volcano plot of 512 differentially expressed genes, where blue and red dots represent DEGs with higher expression levels in the high-*v*_max_ and the low-*v*_max_ pools, respectively. CPM is counts per million. **b** A bubble plot of enriched GO terms, where circle size represents number of enriched genes; and red, blue, and green dots represent biological process, cellular component, and molecular function, respectively. Yellow line is the threshold line with adjusted *P* = 0.05. **c** A relationship network between enriched terms and DEGs. **d** A relationship network between enriched pathways and DEGs

Comparative transcriptomic analysis of the two extreme bulks was performed by RNA sequencing, and the gene expression levels were measured based on FPKM. To simplify the analysis, this study focused only the DEGs located within the mapped QTL regions. We identified a total of 512 QTL-related DEGs, of which 265 genes were expressed at a higher level in the high-*v*_max_ pool and 247 genes were expressed at a higher level in the low-*v*_max_ pool (Fig. 5a). GO analysis assigned these genes to different functional categories, and we found that nine GO terms comprising 17 genes were specifically enriched in the set of DEGs when compared with background frequency. Eight of the nine terms were annotated as involving in “biological process” and one was grouped into the “molecular function” category (Fig. 5b, c). Most represented biological processes were associated with carbohydrate synthesis and metabolism. KEGG analysis of QTL-related DEGs revealed the enrichment of three pathways, including biosynthesis of secondary metabolites (zma01110), starch and sucrose metabolism (zma00500), and taurine and hypotaurine metabolism (zma00430) (Fig. 5d). The three pathways involved 33 DEGs, 10 of which were also identified by GO analysis; as such, a total of 40 genes were revealed by GO and KEGG enrichment analyses.

### Validation of the differentially expressed genes

**Fig. 6:**
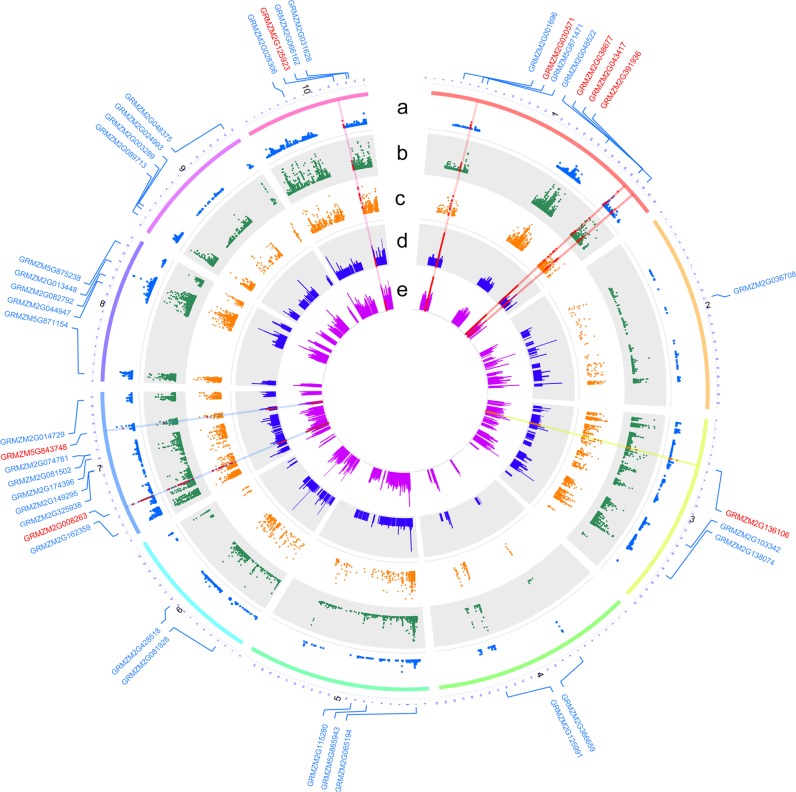
Identification of SNPs associated with the 40 DEGs in the mapped QTL regions. The red dots and bars are statistically significant. The DEGs are indicated around the circle, where DEGs with significantly associated SNP are tagged by red. **a** The −log_10_(*P*_adj_) of each site through Fisher’ exact test. **b** *G* values of individual sites. **c** Absolute values of the ΔSNP index of individual sites. **d** The −log_10_(*P*_adj_) of genes in the QTL regions through differentially expressed analysis. **e** |log_2_(fold change)| of DEGs in the QTL regions. Based on these indicators, eight associated genes with significantly associated SNPs and enriched terms or pathways are identified

In addition to identification of DEGs, the BSR-seq analysis also allowed for detection of allelic variants that were differentially present between pools. These variants could be causally or non-causally associated with the phenotypic differences or differential gene expression. For this study, we were specifically interested in identifying SNPs associated with the 40 DEGs revealed by the GO and KEGG enrichment analyses. Based on Fisher’s exact test, ΔSNP index, and *G* values, we identified eight genes whose allele frequencies differed significantly between the two pools (Fig. 6). These genes included *GRMZM2G030571*, *GRMZM2G038677*, *GRMZM2G043417*, *GRMZM2G391936*, *GRMZM2G136106*, *GRMZM2G008263*, *GRMZM5G843748*, and *GRMZM2G125923*. We assumed that these genes likely play important roles in regulating the seed-filling process.

**Fig. 7:**
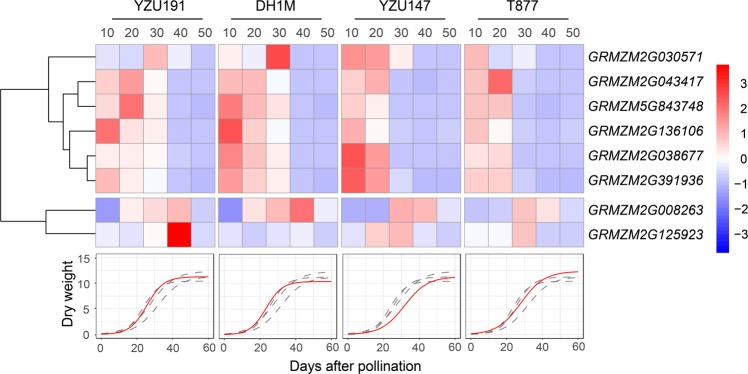
Expression levels of eight related genes in different seed-filling phases of the two parents and RILs. Red color represents relatively higher expression levels and blue color represents relatively lower levels. Red curves represent seed-filling processes of individual inbred lines. Genotypes with similar seed-filling trajectories shared similar expression profiles across different genes

To further validate our assumption, we characterized the expression of these genes in the developing seeds of the two parents T877 and DH1M, as well as two additional inbred lines YZU147 and YZU191, at 10–50 days after pollination (Fig. 7). The filling dynamics was similar between YZU147 and T877 and between YZU191 and DH1M. Cluster analysis classified these genes into two groups based on their expression patterns across genotypes. The first group included genes *GRMZM2G030571*, *GRMZM2G038677*, *GRMZM2G043417*, *GRMZM2G391936*, *GRMZM2G136106*, and *GRMZM5G843748* that were expressed at a higher level at the early filling stage, while the second group contained genes *GRMZM2G008263* and *GRMZM2G125923* that appeared to be expressed at a higher level at the mid-filling stage. Overall, genotypes with similar seed-filling trajectories shared similar expression profiles across different genes, suggesting that changes in the expression levels of these genes are associated with seed filling.

Interestingly, two of the eight genes (*GRMZM2G391936* and *GRMZM2G008263*) are associated with the starch and sucrose metabolism. *GRMZM2G391936* (*agpllzm*) encodes a leaf large subunit of ADP-glucose pyrophosphorylase (AGPase). AGPase is a rate-limiting enzyme (Georgelis et al. 2009) and catalyzes the reaction of glucose-1-phosphate (Glc-1-P) and adenosine triphosphate to produce ADP glucose (ADPGlc) and inorganic pyrophosphate, where ADPGlc formation is an important metabolic control point (Huang et al. 2014). The yield of several starch-producing crops can be increased by altered AGPase activity (Smidansky et al. 2007; Hannah et al. 2012; Tuncel and Okita 2013). Although *GRMZM2G391936* usually has a higher expression level in leaves, some studies showed that this gene was present in maize endosperm after pollination (Davidson et al. 2011; Huang et al. 2014). It has been reported that starch content of homozygous *agpllzm-Ds1* mutant endosperm in maize had a statistically significant reduction when compared with the starch content of wild-type kernels at 20 DAP (Huang et al. 2014). *GRMZM2G008263* (*GBSSIIa*) encodes a granule bound starch synthase that is localized exclusively within the starch granule and is responsible for amylose biosynthesis. *GBSSIIa* also plays a role in the elongation of long chains in amylopectin (Grimaud et al. 2008). The activity of GBSS was significantly and positively correlated with the accumulation rate of starch and its components in maize lines, especially the amylose accumulation rate (Zhang et al. 2008). Therefore, mutation of *GBSSIIa* could result in changes in GBSS activity, greatly influencing amylose accumulation. AGPase catalyzes Glc-1-P to produce ADPGlc, and ADPGlc is an acting substrate of GBSS, indicating that *GRMZM2G008263* plays a role after *GRMZM2G391936* in the starch biosynthesis pathway. In consistent with this, we observed that upregulation of *GRMZM2G391936* occurred before that of *GRMZM2G008263*, and that the enhanced expression of *GRMZM2G008263* lasted until 40 DAP, which was longer than *GRMZM2G391936*. The inbred lines (YZU191 and DH1M) with a higher filling rate exhibited a higher expression level of both *GRMZM2G008263* and *GRMZM2G391936* when compared with the inbred lines (YZU147 and T877) with a lower filling rate. These observations suggest an important role of the two genes in seed filling. Notably, despite we only focused on SNPs in QTL regions, it was still possible that there were linkage blocks. If the causal variant is linked to a gene(s) that has *cis*-controlled expression variation in the parents, and also likely SNPs, this linked gene(s) would still be classified as significant by this assay. In this study, we further verified these candidate genes by the change of expression levels during the filling process. Two of these candidate genes were well known for playing roles in seed filling, which supported our conclusion that the logistic model-based approach can efficiently identify genetic loci that regulate dynamic developmental traits. In addition, due to feedback regulation and post-translational modifications, some potential candidate genes could have no difference in transcriptional level (Schlötterer et al. 2014). Therefore, some potential candidate genes are still unable to be detected in this study. Other methods would be needed to mine these potential candidate genes.

### Summary

Seed filling is a vital process of seed development. Seed-filling rate and duration determine the yield of grain crops, and an efficient and rapid filling process is crucial to achieve high yield. Understanding the genetic factors underlying seed filling will facilitate breeding for optimal filling rate and duration. Seed development follows a sigmoid growth curve, with each phase contributing to the final seed size. Traditional studies have been focused on the linear phase of the seed development and thus failed to capture the dynamic developing features of the filling process. In this study, we employed a logistic function-based approach to model the seed-filling dynamics and applied genetic and genomic tools to search for genetic factors that are associated with the seed-filling process in maize. QTL mapping in an RIL population coupled with bulked segregant RNA-seq analysis allowed for identifying multiple QTL loci and associated genes, including two genes well known for playing roles in seed filling. This study validates our hypothesis that the logistic model-based approach can efficiently identify genetic loci that regulate dynamic developmental traits.

### Data archiving

Seed-filling characteristic parameters of RIL in three environments and genotypic data, as well as the RNA-seq FPKM data sets are available from Figshare (10.6084/m9.figshare.7588547). The raw fastq files were deposited in the Gene Expression Omnibus: accession number GSE130930.

## Supplementary information

Supplemental Material
