# Supplementary material for: Genetic and genomic analysis of the seed-filling process in maize based on a logistic model
Source: Heredity (Edinb). 2019 Jul 29;124(1):122–34. doi: 10.1038/s41437-019-0251-x (PMC6906428; doi:10.1038/s41437-019-0251-x)
Supplement: Supplementary file 1 — Supplemental Material [file 41437_2019_251_MOESM1_ESM.docx]

**Supplementary figures**


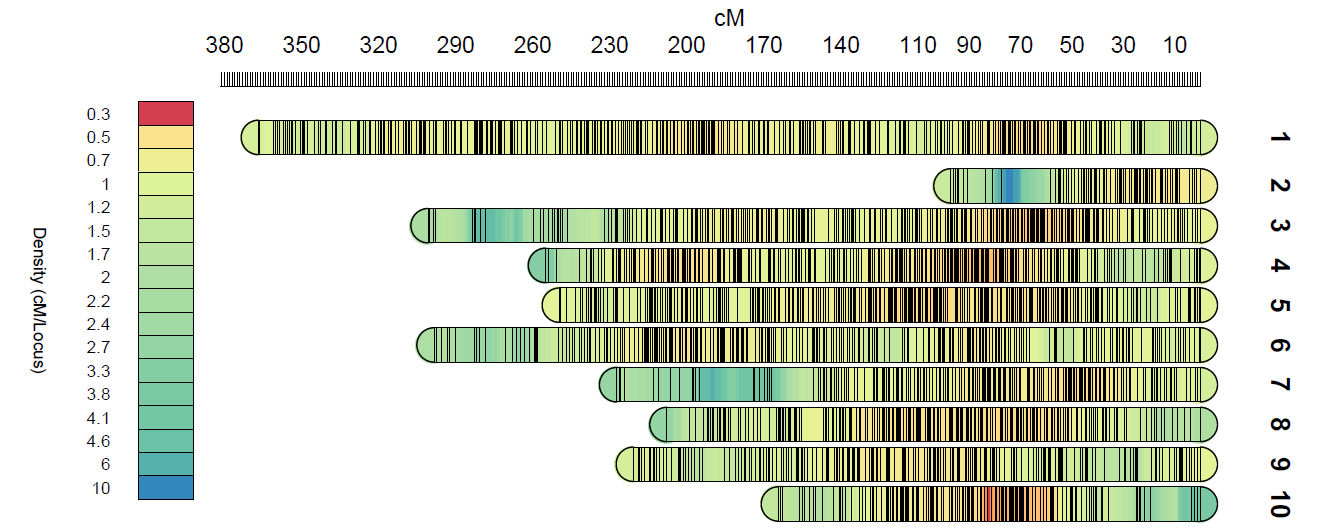


**Figure S1** A high-density genetic linkage map with 3,227 bin markers.


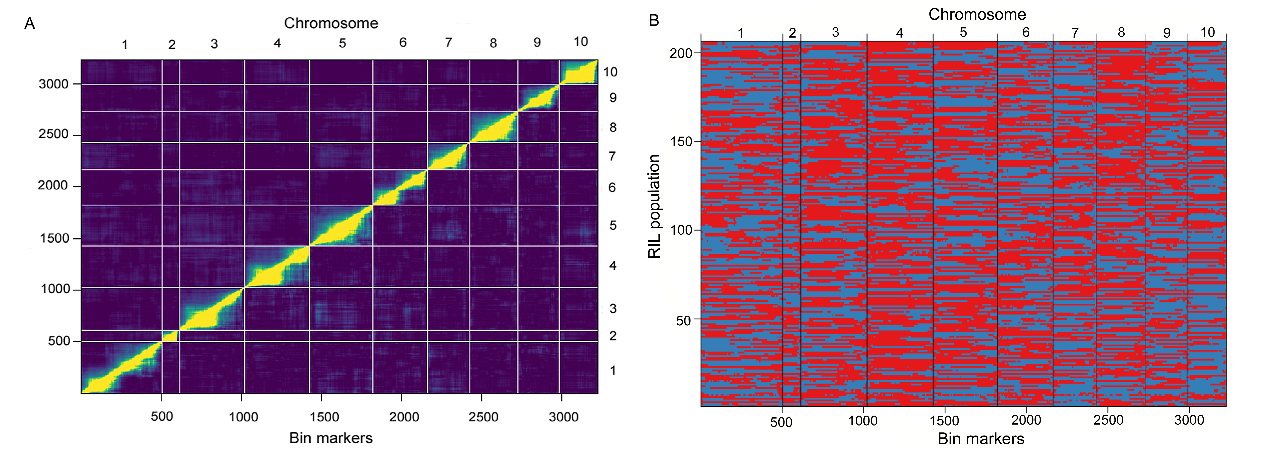


**Figure S2** A heatmap of the linkage and recombination relationship between bin markers and a grid for the haplotypes of the RIL population. (A): Upper left is estimated recombination fractions and lower right is LOD scores for all pairs of markers. Brighter color indicated stronger linkage. (B): Red fragment represents genotype of parent T877, and blue fragment represents genotype of parent DH1M.

**Supplementary tables**

| **Table S1 Phenotypes of RIL population across three environments.** | | | | | | | | | | | |
| --- | --- | --- | --- | --- | --- | --- | --- | --- | --- | --- | --- |
| Parameters | Environments | DH1M | T877 | RIL population | | | | | | | |
|  |  |  |  | Mean | SD | Maximum | Minimum | Skewness | Kurtosis | CV(%) | Heritability (%) |
| *k* | 2015 Nantong | 8.459 | 11.435 | 9.135 | 2.893 | 18.971 | 4.065 | 0.843 | 0.512 | 31.67 | 78.44 |
|  | 2016 Yangzhou | 8.774 | 10.267 | 8.524 | 2.966 | 18.700 | 2.447 | 0.891 | 1.092 | 34.80 |  |
|  | 2017 Sanya | 15.196 | 17.466 | 15.585 | 2.081 | 22.289 | 10.850 | 0.121 | 0.053 | 13.35 |  |
| *b* | 2015 Nantong | 0.195 | 0.140 | 0.211 | 0.095 | 0.833 | 0.080 | 2.83 | 13.324 | 45.11 | 69.26 |
|  | 2016 Yangzhou | 0.238 | 0.175 | 0.173 | 0.074 | 0.574 | 0.069 | 1.966 | 6.518 | 42.65 |  |
|  | 2017 Sanya | 0.144 | 0.112 | 0.135 | 0.027 | 0.243 | 0.056 | 0.787 | 2.333 | 19.97 |  |
| *lna/b* | 2015 Nantong | 24.350 | 31.878 | 25.923 | 4.633 | 37.366 | 15.153 | 0.429 | -0.047 | 17.87 | 75.37 |
|  | 2016 Yangzhou | 26.164 | 30.137 | 29.969 | 5.849 | 47.734 | 15.445 | 0.686 | 0.222 | 19.52 |  |
|  | 2017 Sanya | 30.941 | 36.720 | 31.689 | 3.019 | 50.465 | 25.415 | 1.872 | 8.93 | 9.53 |  |
| *t_1_* | 2015 Nantong | 17.591 | 22.486 | 18.776 | 3.361 | 26.207 | 8.962 | 0.134 | -0.221 | 17.90 | 79.33 |
|  | 2016 Yangzhou | 20.620 | 22.632 | 21.227 | 3.945 | 34.671 | 11.458 | 0.657 | 0.78 | 18.59 |  |
|  | 2017 Sanya | 21.792 | 25.000 | 21.548 | 2.278 | 30.050 | 16.298 | 0.485 | 1.133 | 10.57 |  |
| *t_2_* | 2015 Nantong | 31.109 | 41.269 | 33.071 | 6.596 | 51.530 | 19.481 | 0.57 | 0.338 | 19.95 | 72.64 |
|  | 2016 Yangzhou | 31.708 | 37.642 | 38.710 | 8.535 | 61.799 | 19.432 | 0.682 | 0.022 | 22.05 |  |
|  | 2017 Sanya | 40.090 | 48.440 | 41.830 | 4.732 | 73.973 | 32.192 | 2.311 | 12.5 | 11.31 |  |
| *t_3_* | 2015 Nantong | 47.934 | 64.647 | 50.862 | 12.248 | 92.413 | 24.906 | 0.629 | 0.664 | 24.08 | 70.57 |
|  | 2016 Yangzhou | 45.508 | 56.323 | 60.470 | 15.995 | 109.431 | 29.357 | 0.675 | 0.059 | 26.45 |  |
|  | 2017 Sanya | 62.864 | 77.613 | 67.073 | 9.804 | 132.488 | 46.610 | 2.197 | 11.658 | 14.62 |  |
| *w_1_* | 2015 Nantong | 1.788 | 2.417 | 1.931 | 0.611 | 4.009 | 0.859 | 0.843 | 0.512 | 31.67 | 78.44 |
|  | 2016 Yangzhou | 1.854 | 2.170 | 1.801 | 0.627 | 3.952 | 0.517 | 0.891 | 1.092 | 34.80 |  |
|  | 2017 Sanya | 3.211 | 3.691 | 3.293 | 0.440 | 4.710 | 2.293 | 0.121 | 0.053 | 13.35 |  |
| *w_2_* | 2015 Nantong | 4.884 | 6.602 | 5.274 | 1.670 | 10.953 | 2.347 | 0.843 | 0.512 | 31.67 | 78.44 |
|  | 2016 Yangzhou | 5.066 | 5.928 | 4.921 | 1.712 | 10.796 | 1.413 | 0.891 | 1.092 | 34.80 |  |
|  | 2017 Sanya | 8.773 | 10.084 | 8.998 | 1.201 | 12.868 | 6.264 | 0.121 | 0.053 | 13.35 |  |
| *w_3_* | 2015 Nantong | 1.703 | 2.302 | 1.839 | 0.583 | 3.819 | 0.818 | 0.843 | 0.512 | 31.67 | 78.44 |
|  | 2016 Yangzhou | 1.767 | 2.067 | 1.716 | 0.597 | 3.765 | 0.493 | 0.891 | 1.092 | 34.80 |  |
|  | 2017 Sanya | 3.059 | 3.516 | 3.138 | 0.419 | 4.487 | 2.184 | 0.121 | 0.053 | 13.35 |  |
| *$\bar{v}$* | 2015 Nantong | 0.275 | 0.267 | 0.300 | 0.105 | 0.912 | 0.121 | 1.79 | 7.225 | 34.90 | 69.4 |
|  | 2016 Yangzhou | 0.347 | 0.300 | 0.228 | 0.080 | 0.621 | 0.076 | 1.595 | 4.957 | 35.10 |  |
|  | 2017 Sanya | 0.365 | 0.327 | 0.349 | 0.076 | 0.631 | 0.163 | 0.63 | 1.341 | 21.77 |  |
| *T* | 2015 Nantong | 30.794 | 42.788 | 32.563 | 11.106 | 75.084 | 7.204 | 0.585 | 0.953 | 34.11 | 73.12 |
|  | 2016 Yangzhou | 25.258 | 34.193 | 39.827 | 14.403 | 87.179 | 10.456 | 0.65 | 0.302 | 36.16 |  |
|  | 2017 Sanya | 41.684 | 53.395 | 46.202 | 9.858 | 107.100 | 24.675 | 1.779 | 8.582 | 21.34 |  |
| *v_max_* | 2015 Nantong | 0.412 | 0.401 | 0.450 | 0.157 | 1.368 | 0.182 | 1.79 | 7.225 | 34.90 | 73.12 |
|  | 2016 Yangzhou | 0.521 | 0.450 | 0.342 | 0.120 | 0.932 | 0.114 | 1.595 | 4.957 | 35.10 |  |
|  | 2017 Sanya | 0.547 | 0.491 | 0.524 | 0.114 | 0.947 | 0.245 | 0.63 | 1.341 | 21.77 |  |

| **Table S2 Summary statistics of the genetic linkage map for the RIL population in this study.** | | | | |
| --- | --- | --- | --- | --- |
| Chromosome | Number of bins | Chromosome size (cM) | Average gap (cM) | Maximum gap (cM) |
| 1 | 503 | 373.06 | 0.743 | 10.74 |
| 2 | 111 | 102.29 | 0.93 | 23.02 |
| 3 | 406 | 311.63 | 0.769 | 22.22 |
| 4 | 406 | 259.27 | 0.64 | 9.61 |
| 5 | 397 | 253.16 | 0.639 | 5.65 |
| 6 | 340 | 305.06 | 0.9 | 8.44 |
| 7 | 264 | 238.37 | 0.906 | 25.39 |
| 8 | 301 | 212.06 | 0.707 | 9.59 |
| 9 | 258 | 225.12 | 0.876 | 9.02 |
| 10 | 241 | 170.28 | 0.71 | 18.61 |
| Total | 3227 | 2450.31 | 0.762 | 25.39 |

| **Table S3 The summary of detected QTLs in different environments**. | | | | | | | | | |
| --- | --- | --- | --- | --- | --- | --- | --- | --- | --- |
| Characteristic parameter | | No. of QTLs | | | | | PVE(%) | | |
|  |  | 2015 Nantong | 2016 Yangzhou | 2017 Sanya | Average | Total  (non-redundant) | Mean | Minimum | Maximum |
| associated with dry weight | *k* | 4 | 2 | 1 | 3 | 10 | 5.54 | 1.94 | 16.27 |
|  | *w_1_* | 4 | 3 | 1 | 3 | 11 | 5.60 | 2.11 | 16.27 |
|  | *w_2_* | 4 | 3 | 1 | 2 | 9 | 5.65 | 1.94 | 14.48 |
|  | *w_3_* | 4 | 3 | 1 | 3 | 10 | 5.23 | 1.54 | 16.27 |
|  | Total  (non-redundant) | 5 | 3 | 1 | 4 | 12 | 5.46 | 1.54 | 16.27 |
| associated with filling duration | *lna/b* | 5 | 7 | 2 | 5 | 19 | 4.89 | 1.25 | 10.42 |
|  | *t_1_* | 4 | 3 | 2 | 4 | 12 | 6.26 | 1.46 | 18.55 |
|  | *t_2_* | 3 | 8 | 3 | 1 | 14 | 6.29 | 1.57 | 11.95 |
|  | *t_3_* | 4 | 4 | 4 | 0 | 12 | 5.10 | 1.49 | 9.46 |
|  | *T* | 4 | 7 | 4 | 0 | 15 | 5.49 | 3.12 | 9.10 |
|  | Total  (non-redundant) | 15 | 23 | 14 | 10 | 60 | 5.57 | 1.25 | 18.55 |
| associated with filling rate | *b* | 2 | 2 | 6 | 0 | 10 | 5.88 | 1.86 | 11.44 |
|  | 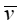 | 2 | 4 | 4 | 5 | 15 | 4.59 | 1.09 | 9.29 |
|  | *v_max_* | 2 | 4 | 4 | 5 | 15 | 4.59 | 1.09 | 9.29 |
|  | Total  (non-redundant) | 4 | 6 | 8 | 5 | 23 | 5.02 | 1.09 | 11.44 |
| Total(non-redundant) | | 22 | 29 | 23 | 19 | 90 | 5.39 | 1.09 | 18.55 |

| **Table S4 The detected QTLs related characteristic parameters in different environments.** | | | | | | | | | | | |
| --- | --- | --- | --- | --- | --- | --- | --- | --- | --- | --- | --- |
| ID | Non-redundant QTL | Environment | Characteristic Parameter | Chromosome | Peak marker | LOD | Additive effect | R^2^(%) | Genetic position(cM) | Physical Positon(bp) | Bin size (Mbp) |
| 1 | 1 | 2015 Nantong | ** | 10 | SYNGENTA3628 | 5.42 | -0.033 | 9.27 | 35.63 | 5878157 | 2.68 |
| 2 | 1 | 2015 Nantong | *v_max_* | 10 | SYNGENTA3628 | 5.42 | -0.049 | 9.27 | 35.63 | 5878157 | 2.68 |
| 3 | 2 | 2016 Yangzhou | *k* | 7 | SYN39338 | 4.05 | 0.67 | 3.31 | 14.09 | 9713438 | 2.75 |
| 4 | 2 | 2016 Yangzhou | *t_2_* | 7 | SYN39338 | 4.06 | 1.78 | 1.58 | 14.09 | 9713438 | 2.75 |
| 5 | 2 | 2016 Yangzhou | *w_1_* | 7 | SYN39338 | 4.05 | 0.142 | 3.31 | 14.09 | 9713438 | 2.75 |
| 6 | 2 | 2016 Yangzhou | *w_2_* | 7 | SYN39338 | 4.05 | 0.387 | 3.31 | 14.09 | 9713438 | 2.75 |
| 7 | 2 | 2016 Yangzhou | *w_3_* | 7 | SYN39338 | 4.05 | 0.135 | 3.31 | 14.09 | 9713438 | 2.75 |
| 8 | 3 | 2016 Yangzhou | *w_1_* | 10 | PZE-110018165 | 3.73 | 0.267 | 5.33 | 68.71 | 21403652 | 2.35 |
| 9 | 3 | 2016 Yangzhou | *w_2_* | 10 | PZE-110018165 | 3.73 | 0.728 | 5.33 | 68.71 | 21403652 | 2.35 |
| 10 | 3 | 2016 Yangzhou | *w_3_* | 10 | PZE-110018165 | 3.73 | 0.254 | 5.33 | 68.71 | 21403652 | 2.35 |
| 11 | 4 | average | ** | 6 | PZE-106009740 | 2.88 | 0.001 | 4.21 | 30.39 | 27500630 | 2.66 |
| 12 | 4 | average | *v_max_* | 6 | PZE-106009740 | 2.88 | 0.002 | 4.21 | 30.39 | 27500630 | 2.66 |
| 13 | 5 | 2015 Nantong | *T* | 5 | PZE-105069274 | 3.5 | 2.565 | 4.47 | 95.38 | 73067453 | 1 |
| 14 | 5 | 2015 Nantong | *t_3_* | 5 | PZE-105069274 | 4.68 | 3.19 | 5.43 | 95.38 | 73067453 | 1 |
| 15 | 6 | 2016 Yangzhou | ** | 3 | PZE-103115131 | 5.3 | 0.024 | 4 | 150.11 | 175143570 | 2.3 |
| 16 | 6 | 2016 Yangzhou | *v_max_* | 3 | PZE-103115131 | 5.3 | 0.035 | 4 | 150.11 | 175143570 | 2.3 |
| 17 | 7 | average | ** | 3 | PZE-103108109 | 4.16 | -0.001 | 2.02 | 135.53 | 169087927 | 1.38 |
| 18 | 7 | average | *v_max_* | 3 | PZE-103108109 | 4.16 | -0.001 | 2.02 | 135.53 | 169087927 | 1.38 |
| 19 | 8 | average | ** | 1 | PZE-101242296 | 7.5 | 0.002 | 9.29 | 349 | 288833364 | 2.81 |
| 20 | 8 | average | *v_max_* | 1 | PZE-101242296 | 7.5 | 0.003 | 9.29 | 349 | 288833364 | 2.81 |
| 21 | 9 | average | ** | 1 | PZE-101148225 | 3.32 | 0.001 | 1.85 | 136 | 191886324 | 1.39 |
| 22 | 9 | average | *v_max_* | 1 | PZE-101148225 | 3.32 | 0.001 | 1.85 | 136 | 191886324 | 1.39 |
| 23 | 10 | 2015 Nantong | *b* | 6 | SYN29969 | 3.89 | -0.023 | 5.3 | 230.7 | 150268512 | 1.41 |
| 24 | 10 | 2015 Nantong | *lna/b* | 6 | SYN29969 | 3.34 | 1.923 | 7.2 | 230.7 | 150268512 | 1.41 |
| 25 | 10 | 2015 Nantong | *t_2_* | 6 | SYN29969 | 5.95 | 2.143 | 9.46 | 230.7 | 150268512 | 1.41 |
| 26 | 11 | 2017 Sanya | ** | 1 | SYN2732 | 3.32 | -0.018 | 3.05 | 318.2 | 279642905 | 1.95 |
| 27 | 11 | 2017 Sanya | *v_max_* | 1 | SYN2732 | 3.32 | -0.027 | 3.05 | 318.2 | 279642905 | 1.95 |
| 28 | 12 | 2015 Nantong | *k* | 4 | SYN23104 | 5.54 | 0.631 | 3.6 | 129.41 | 167216340 | 2.78 |
| 29 | 12 | 2015 Nantong | *w_1_* | 4 | SYN23104 | 5.54 | 0.133 | 3.6 | 129.41 | 167216340 | 2.78 |
| 30 | 13 | 2016 Yangzhou | ** | 5 | SYN16506 | 4.79 | 0.029 | 6.52 | 32.74 | 17399413 | 2.1 |
| 31 | 13 | 2016 Yangzhou | *v_max_* | 5 | SYN16506 | 4.79 | 0.043 | 6.52 | 32.74 | 17399413 | 2.1 |
| 32 | 14 | 2017 Sanya | *b* | 8 | PZE-108010981 | 2.98 | -0.004 | 2.35 | 26 | 11537594 | 3.12 |
| 33 | 14 | 2017 Sanya | ** | 8 | PZE-108010981 | 4.21 | -0.018 | 3.71 | 28.67 | 11537594 | 3.12 |
| 34 | 14 | 2017 Sanya | *v_max_* | 8 | PZE-108010981 | 4.21 | -0.028 | 3.71 | 28.67 | 11537594 | 3.12 |
| 35 | 15 | 2015 Nantong | *k* | 8 | PZE-108007014 | 3.47 | -0.535 | 2.9 | 8.74 | 7039901 | 3 |
| 36 | 15 | 2015 Nantong | *w_1_* | 8 | PZE-108007014 | 3.47 | -0.113 | 2.9 | 8.74 | 7039901 | 3 |
| 37 | 15 | 2015 Nantong | *w_2_* | 8 | PZE-108007014 | 3.47 | -0.292 | 2.62 | 8.74 | 7039901 | 3 |
| 38 | 15 | 2015 Nantong | *w_3_* | 8 | PZE-108007014 | 3.47 | -0.108 | 2.9 | 8.74 | 7039901 | 3 |
| 39 | 16 | 2017 Sanya | *b* | 4 | PZE-104093451 | 5.02 | 0.009 | 8.44 | 135.24 | 170004831 | 8.67 |
| 40 | 16 | 2017 Sanya | ** | 4 | PZE-104093451 | 7.8 | 0.017 | 2.47 | 135 | 170004831 | 8.67 |
| 41 | 16 | 2017 Sanya | *v_max_* | 4 | PZE-104093451 | 7.8 | 0.025 | 2.47 | 135 | 170004831 | 8.67 |
| 42 | 17 | 2015 Nantong | ** | 3 | PZE-103113658 | 3.57 | -0.017 | 2.27 | 144 | 173416786 | 3.67 |
| 43 | 17 | 2015 Nantong | *v_max_* | 3 | PZE-103113658 | 3.57 | -0.025 | 2.27 | 144 | 173416786 | 3.67 |
| 44 | 18 | 2016 Yangzhou | *k* | 8 | SYNGENTA6461 | 3.56 | -0.828 | 4.69 | 179.92 | 164900924 | 1.34 |
| 45 | 18 | 2016 Yangzhou | *t_2_* | 8 | SYNGENTA6461 | 4.53 | -1.562 | 1.84 | 179.92 | 164900924 | 1.34 |
| 46 | 18 | 2016 Yangzhou | *w_1_* | 8 | SYNGENTA6461 | 3.56 | -0.175 | 4.69 | 179.92 | 164900924 | 1.34 |
| 47 | 18 | 2016 Yangzhou | *w_2_* | 8 | SYNGENTA6461 | 3.56 | -0.478 | 4.69 | 179.92 | 164900924 | 1.34 |
| 48 | 18 | 2016 Yangzhou | *w_3_* | 8 | SYNGENTA6461 | 3.56 | -0.167 | 4.69 | 179.92 | 164900924 | 1.34 |
| 49 | 19 | 2015 Nantong | *k* | 2 | SYN7780 | 3.04 | -1.268 | 16.27 | 56.33 | 175621930 | 3.38 |
| 50 | 19 | 2015 Nantong | *T* | 2 | SYN7780 | 3.35 | -2.61 | 4.72 | 56.33 | 175621930 | 3.38 |
| 51 | 19 | 2015 Nantong | *t_2_* | 2 | SYN7780 | 4.6 | -2.09 | 8.45 | 56.33 | 175621930 | 3.38 |
| 52 | 19 | 2015 Nantong | *t_3_* | 2 | SYN7780 | 3.17 | -3.018 | 5.26 | 56.33 | 175621930 | 3.38 |
| 53 | 19 | 2015 Nantong | *w_1_* | 2 | SYN7780 | 3.04 | -0.268 | 16.27 | 56.33 | 175621930 | 3.38 |
| 54 | 19 | 2015 Nantong | *w_2_* | 2 | SYN7780 | 3.04 | -0.681 | 14.48 | 56.33 | 175621930 | 3.38 |
| 55 | 19 | 2015 Nantong | *w_3_* | 2 | SYN7780 | 3.04 | -0.255 | 16.27 | 56.33 | 175621930 | 3.38 |
| 56 | 20 | 2017 Sanya | *lna/b* | 1 | SYN469 | 3 | 0.551 | 2.92 | 0 | 33325943 | 4.08 |
| 57 | 20 | 2017 Sanya | *t_3_* | 1 | SYN469 | 3.24 | 2.057 | 3.65 | 0 | 33325943 | 4.08 |
| 58 | 21 | 2016 Yangzhou | *lna/b* | 1 | SYN38393 | 4.23 | 2.859 | 8.94 | 12.05 | 41541396 | 5.6 |
| 59 | 21 | 2016 Yangzhou | *T* | 1 | SYN38393 | 6.36 | 5.48 | 7.66 | 12.05 | 41541396 | 5.6 |
| 60 | 21 | 2016 Yangzhou | *t_2_* | 1 | SYN38393 | 6.07 | 4.873 | 11.95 | 12.05 | 41541396 | 5.6 |
| 61 | 21 | average | *t_2_* | 1 | SYN38393 | 2.88 | 0.033 | 7.43 | 12.05 | 41541396 | 5.6 |
| 62 | 21 | 2016 Yangzhou | *t_3_* | 1 | SYN38393 | 5.18 | 6.703 | 9.19 | 12.05 | 41541396 | 5.6 |
| 63 | 22 | average | \|  \| \| --- \| | 2 | SYN36647 | 3.94 | -0.001 | 6.4 | 39.33 | 145515791 | 13.71 |
| 64 | 22 | average | *v_max_* | 2 | SYN36647 | 3.94 | -0.002 | 6.4 | 39.33 | 145515791 | 13.71 |
| 65 | 23 | 2016 Yangzhou | *lna/b* | 2 | SYN20472 | 4 | -1.532 | 3.72 | 97.11 | 203708390 | 6.16 |
| 66 | 23 | 2016 Yangzhou | *t_1_* | 2 | SYN20472 | 3.58 | -1.641 | 11.71 | 97.11 | 203708390 | 6.16 |
| 67 | 23 | 2016 Yangzhou | *t_2_* | 2 | SYN20472 | 3.41 | -1.433 | 1.57 | 97.11 | 203708390 | 6.16 |
| 68 | 24 | 2016 Yangzhou | ** | 10 | PZE-110093304 | 4.92 | -0.027 | 8.38 | 156 | 140722907 | 1.83 |
| 69 | 24 | 2016 Yangzhou | *v_max_* | 10 | PZE-110093304 | 4.92 | -0.04 | 8.38 | 156 | 140722907 | 1.83 |
| 70 | 25 | average | *k* | 9 | PZE-109041853 | 3.25 | 0.067 | 1.94 | 97.17 | 66984161 | 6.86 |
| 71 | 25 | average | *w_1_* | 9 | PZE-109041853 | 3.25 | 0.015 | 2.14 | 97.17 | 66984161 | 6.86 |
| 72 | 25 | average | *w_2_* | 9 | PZE-109041853 | 3.25 | 0.039 | 1.94 | 97.17 | 66984161 | 6.86 |
| 73 | 25 | average | *w_3_* | 9 | PZE-109041853 | 3.25 | 0.012 | 1.54 | 97.17 | 66984161 | 6.86 |
| 74 | 26 | 2017 Sanya | *k* | 5 | PZE-105027956 | 2.99 | -0.647 | 7.28 | 24 | 14485994 | 2.78 |
| 75 | 26 | 2017 Sanya | *w_1_* | 5 | PZE-105027956 | 2.99 | -0.137 | 7.28 | 24 | 14485994 | 2.78 |
| 76 | 26 | 2017 Sanya | *w_2_* | 5 | PZE-105027956 | 2.99 | -0.374 | 7.28 | 24 | 14485994 | 2.78 |
| 77 | 26 | 2017 Sanya | *w_3_* | 5 | PZE-105027956 | 2.99 | -0.13 | 7.28 | 24 | 14485994 | 2.78 |
| 78 | 27 | 2017 Sanya | ** | 1 | PZE-101044692 | 3.66 | -0.01 | 1.09 | 1 | 30572534 | 8.87 |
| 79 | 27 | 2017 Sanya | *v_max_* | 1 | PZE-101044692 | 3.66 | -0.015 | 1.09 | 1 | 30572534 | 8.87 |
| 80 | 28 | 2015 Nantong | *lna/b* | 8 | SYN3623 | 3.24 | 1.379 | 6.6 | 93.48 | 110719657 | 2.63 |
| 81 | 28 | 2015 Nantong | *t_1_* | 8 | SYN3623 | 5.88 | 1.837 | 18.55 | 93.48 | 110719657 | 2.63 |
| 82 | 29 | average | *w_1_* | 2 | SYN33703 | 2.99 | -0.012 | 2.11 | 22.34 | 72417245 | 26.92 |
| 83 | 29 | average | *w_3_* | 2 | SYN33703 | 2.99 | -0.011 | 1.9 | 22.34 | 72417245 | 26.92 |
| 84 | 30 | 2015 Nantong | *k* | 9 | PZE-109015720 | 3.86 | 0.743 | 5.45 | 59.29 | 15983535 | 3.18 |
| 85 | 30 | 2015 Nantong | *w_1_* | 9 | PZE-109015720 | 3.86 | 0.157 | 5.45 | 59.29 | 15983535 | 3.18 |
| 86 | 30 | 2015 Nantong | *w_2_* | 9 | PZE-109015720 | 3.86 | 0.41 | 4.98 | 59.29 | 15983535 | 3.18 |
| 87 | 30 | 2015 Nantong | *w_3_* | 9 | PZE-109015720 | 3.86 | 0.15 | 5.45 | 59.29 | 15983535 | 3.18 |
| 88 | 31 | 2016 Yangzhou | *b* | 5 | PZE-105027872 | 3.04 | 0.028 | 11.44 | 23.31 | 14391704 | 1.77 |
| 89 | 31 | 2016 Yangzhou | *T* | 5 | PZE-105027872 | 3.59 | -4.438 | 8.01 | 23.31 | 14391704 | 1.77 |
| 90 | 31 | 2016 Yangzhou | *t_3_* | 5 | PZE-105027872 | 3.34 | -2.235 | 1.49 | 23.31 | 14391704 | 1.77 |
| 91 | 32 | 2015 Nantong | *t_1_* | 1 | PZE-101152711 | 3 | 0.645 | 1.67 | 146.82 | 196161029 | 2.65 |
| 92 | 32 | average | *t_1_* | 1 | PZE-101152711 | 7.42 | 0.174 | 8.62 | 146.82 | 196161029 | 2.65 |
| 93 | 33 | average | *k* | 4 | PZE-104092635 | 4.83 | 0.089 | 4.6 | 129.41 | 168598027 | 1.41 |
| 94 | 33 | average | *w_1_* | 4 | PZE-104092635 | 4.83 | 0.024 | 8.49 | 129.41 | 168598027 | 1.41 |
| 95 | 33 | 2015 Nantong | *w_2_* | 4 | PZE-104092635 | 5.54 | 0.443 | 6.23 | 129.41 | 168598027 | 1.41 |
| 96 | 33 | average | *w_2_* | 4 | PZE-104092635 | 4.83 | 0.051 | 4.6 | 129.41 | 168598027 | 1.41 |
| 97 | 33 | 2015 Nantong | *w_3_* | 4 | PZE-104092635 | 5.54 | 0.127 | 3.6 | 129.41 | 168598027 | 1.41 |
| 98 | 33 | average | *w_3_* | 4 | PZE-104092635 | 4.83 | 0.028 | 7.1 | 129.41 | 168598027 | 1.41 |
| 99 | 34 | 2016 Yangzhou | ** | 6 | PZE-106044272 | 3.25 | -0.02 | 4.28 | 115.12 | 65096023 | 1.95 |
| 100 | 34 | 2016 Yangzhou | *v_max_* | 6 | PZE-106044272 | 3.25 | -0.03 | 4.28 | 115.12 | 65096023 | 1.95 |
| 101 | 35 | 2015 Nantong | *T* | 10 | SYN16747 | 3.16 | 2.39 | 3.73 | 29 | 5594488 | 0.99 |
| 102 | 36 | 2017 Sanya | *b* | 1 | SYN34303 | 3.98 | -0.004 | 1.86 | 1.45 | 30690912 | 6.25 |
| 103 | 37 | 2016 Yangzhou | *t_3_* | 7 | PZE-107015358 | 3.29 | 7.524 | 9.46 | 22 | 12463462 | 2.75 |
| 104 | 38 | 2015 Nantong | *t_3_* | 6 | PZE-106093812 | 3.39 | 2.636 | 3.86 | 229.73 | 149546302 | 1.54 |
| 105 | 39 | 2017 Sanya | *t_3_* | 7 | PZE-107027810 | 5.54 | 3.043 | 7.47 | 43 | 33066748 | 2.72 |
| 106 | 40 | 2015 Nantong | *t_3_* | 10 | PZE-110006344 | 3.24 | 2.237 | 3.06 | 24.31 | 4881219 | 1.03 |
| 107 | 41 | 2017 Sanya | *t_3_* | 3 | PZE-103087178 | 3.1 | 2.321 | 2.73 | 93.01 | 144717717 | 1.67 |
| 108 | 42 | 2016 Yangzhou | *t_3_* | 9 | PZE-109020307 | 3.58 | -5.875 | 4.63 | 72 | 20596316 | 1.75 |
| 109 | 43 | 2017 Sanya | *t_3_* | 8 | PZE-108108630 | 4.52 | 3.086 | 5.02 | 164.38 | 161724378 | 1.88 |
| 110 | 44 | 2016 Yangzhou | *t_2_* | 9 | sh1.1 | 4.93 | 2.157 | 3.09 | 37 | 11505493 | 1.69 |
| 111 | 45 | 2017 Sanya | *t_2_* | 8 | PZE-108110136 | 7.38 | 1.599 | 9.59 | 167 | 162552560 | 2.23 |
| 112 | 46 | 2017 Sanya | *t_2_* | 7 | PZE-107028398 | 3.93 | 1.239 | 5.32 | 43.22 | 33962755 | 4.95 |
| 113 | 47 | 2017 Sanya | *t_2_* | 3 | PZE-103154530 | 4.56 | -1.889 | 8.86 | 210.32 | 207499848 | 1.54 |
| 114 | 48 | 2016 Yangzhou | *t_2_* | 5 | PZE-105053157 | 3.77 | 2.199 | 3.28 | 75.47 | 48709447 | 1.41 |
| 115 | 49 | 2016 Yangzhou | *t_2_* | 4 | SYN15646 | 6.93 | -4.899 | 9.27 | 160 | 183761350 | 2.13 |
| 116 | 50 | 2015 Nantong | *t_2_* | 3 | PZE-103017733 | 4.15 | -1.93 | 6.45 | 10 | 10391494 | 1.74 |
| 117 | 51 | 2016 Yangzhou | *t_2_* | 6 | PZE-106036046 | 5.15 | 2.51 | 6.2 | 96.18 | 83774147 | 2.47 |
| 118 | 52 | average | *t_1_* | 5 | PZE-105040202 | 4.3 | 0.133 | 3.88 | 55.56 | 25564076 | 3.41 |
| 119 | 53 | 2017 Sanya | *t_1_* | 8 | PZE-108108785 | 5.46 | 0.618 | 5.9 | 165 | 161741863 | 1.99 |
| 120 | 54 | 2015 Nantong | *t_1_* | 6 | PZE-106009322 | 3.48 | 2.295 | 7.2 | 28.93 | 26708916 | 2.64 |
| 121 | 55 | average | *t_1_* | 6 | SYN21064 | 3.27 | 0.097 | 2.08 | 6 | 3777500 | 2.48 |
| 122 | 56 | 2016 Yangzhou | *t_1_* | 10 | PZE-110064304 | 3.6 | 0.822 | 1.95 | 104.27 | 119975333 | 2.26 |
| 123 | 57 | 2016 Yangzhou | *t_1_* | 1 | SYN36487 | 3.61 | 1.306 | 6.42 | 5 | 36218643 | 5.95 |
| 124 | 58 | 2017 Sanya | *t_1_* | 6 | SYN27126 | 3.05 | -0.56 | 5.29 | 282 | 159967832 | 1.88 |
| 125 | 59 | 2015 Nantong | *t_1_* | 5 | PZE-105078104 | 3.24 | 1.005 | 6.69 | 104.12 | 88263965 | 3.11 |
| 126 | 60 | 2015 Nantong | *T* | 6 | PZE-106094423 | 3.46 | 2.995 | 6.93 | 229.73 | 149873980 | 1.8 |
| 127 | 61 | 2016 Yangzhou | *T* | 6 | PZE-106034370 | 4.1 | 3.178 | 4.28 | 91 | 80799717 | 3.3 |
| 128 | 62 | 2017 Sanya | *T* | 3 | PZE-103086995 | 3.78 | 2.713 | 3.83 | 93.01 | 144128249 | 2.15 |
| 129 | 63 | 2016 Yangzhou | *T* | 9 | PZE-109107501 | 2.98 | -4.293 | 7.1 | 210 | 149387498 | 1.8 |
| 130 | 64 | 2017 Sanya | *T* | 7 | PZE-107027417 | 4.63 | 3.116 | 9.1 | 42 | 32258870 | 1.93 |
| 131 | 65 | 2016 Yangzhou | *T* | 3 | PZE-103089288 | 3.73 | 5.064 | 4.3 | 98 | 147630346 | 0.81 |
| 132 | 66 | 2016 Yangzhou | *T* | 7 | PZE-107128048 | 3.02 | -3.418 | 4.5 | 202 | 170438672 | 2.35 |
| 133 | 67 | 2017 Sanya | *T* | 9 | SYN30121 | 4.79 | 2.634 | 4.1 | 195.12 | 146724089 | 1.57 |
| 134 | 68 | 2016 Yangzhou | *T* | 10 | PZE-110095259 | 4.05 | 4.054 | 6.53 | 161 | 141705554 | 2.25 |
| 135 | 69 | 2017 Sanya | *T* | 8 | PZE-108110343 | 3.4 | 1.955 | 3.12 | 169 | 162770443 | 2.18 |
| 136 | 70 | 2016 Yangzhou | *lna/b* | 9 | PZE-109014671 | 4.17 | 1.57 | 2.59 | 55.41 | 14583733 | 4.05 |
| 137 | 71 | average | *lna/b* | 8 | PZE-108079027 | 4.28 | 0.062 | 1.71 | 122.4 | 134593696 | 1.99 |
| 138 | 72 | 2016 Yangzhou | *lna/b* | 4 | PZE-104106680 | 6.09 | -3.094 | 8.75 | 157.09 | 183205963 | 1.81 |
| 139 | 73 | 2015 Nantong | *lna/b* | 4 | PZE-104089707 | 4.59 | 1.875 | 10.42 | 127.47 | 165694100 | 3.08 |
| 140 | 74 | average | *lna/b* | 3 | PZE-103044795 | 3.4 | 0.058 | 2.7 | 56.6 | 45067139 | 2.26 |
| 141 | 75 | 2015 Nantong | *lna/b* | 5 | PZE-105078645 | 2.82 | 1.491 | 7.48 | 105 | 89360472 | 2.49 |
| 142 | 76 | 2015 Nantong | *lna/b* | 3 | PZE-103018067 | 3.2 | -1.51 | 4.18 | 11 | 10517261 | 1.65 |
| 143 | 77 | average | *lna/b* | 1 | SYN38577 | 5.35 | -0.059 | 3.09 | 186.38 | 213026885 | 3.2 |
| 144 | 78 | average | *lna/b* | 7 | PZE-107125846 | 2.98 | -0.08 | 4.55 | 191 | 168634589 | 2.06 |
| 145 | 79 | 2016 Yangzhou | *lna/b* | 3 | SYN16511 | 7.57 | 3.58 | 9.22 | 98.35 | 147790067 | 1.75 |
| 146 | 80 | 2016 Yangzhou | *lna/b* | 6 | SYN12838 | 4.25 | 1.498 | 2.45 | 102 | 87286273 | 1.59 |
| 147 | 81 | 2016 Yangzhou | *lna/b* | 10 | PZE-110066918 | 3.04 | 1.037 | 1.25 | 107.67 | 123390127 | 3.21 |
| 148 | 82 | 2017 Sanya | *lna/b* | 3 | PZE-103026244 | 3.91 | -0.669 | 2.36 | 35.24 | 19264421 | 1.14 |
| 149 | 83 | average | *lna/b* | 5 | PZE-105021345 | 4.35 | 0.059 | 2.78 | 1.93 | 10184109 | 1.17 |
| 150 | 84 | average | *k* | 10 | PZE-110019581 | 3.73 | 1.262 | 5.33 | 69.68 | 24447253 | 1.12 |
| 151 | 85 | 2015 Nantong | *b* | 5 | PZE-105073088 | 4.83 | -0.028 | 6.22 | 100.23 | 78415600 | 4.52 |
| 152 | 86 | 2017 Sanya | *b* | 3 | PZE-103025333 | 2.96 | 0.006 | 3.33 | 31.35 | 18013572 | 1.94 |
| 153 | 87 | 2017 Sanya | *b* | 7 | PZE-107072354 | 5.47 | -0.009 | 7.7 | 83.52 | 128680536 | 1.59 |
| 154 | 88 | 2017 Sanya | *b* | 10 | PZE-110008930 | 3.09 | 0.008 | 6.88 | 42.48 | 6660268 | 2.84 |
| 155 | 89 | average | *t_1_* | 4 | PZE-104011306 | 3.71 | 0.367 | 1.46 | 26.13 | 9866197 | 0.22 |
| 156 | 90 | 2016 Yangzhou | *b* | 3 | PZE-103026136 | 3.73 | -0.018 | 5.28 | 34.27 | 19174016 | 1.17 |
